# Characterization of METRNβ as a novel biomarker of Coronavirus disease 2019 severity and prognosis

**DOI:** 10.3389/fimmu.2023.1111920

**Published:** 2023-01-31

**Authors:** Xun Gao, Paul Kay-Sheung Chan, Katie Ching-Yau Wong, Rita Wai-Yin Ng, Apple Chung-Man Yeung, Grace Chung-Yan Lui, Lowell Ling, David Shu-Cheong Hui, Danqi Huang, Chun-Kwok Wong

**Affiliations:** ^1^ Center of Clinical Laboratory Medicine, Zhongda Hospital, Southeast University, Nanjing, China; ^2^ Department of Chemical Pathology, The Chinese University of Hong Kong, Hong Kong, Hong Kong SAR, China; ^3^ Department of Microbiology, The Chinese University of Hong Kong, Hong Kong, Hong Kong SAR, China; ^4^ Stanley Ho Centre for Emerging Infectious Diseases, The Chinese University of Hong Kong, Hong Kong, Hong Kong SAR, China; ^5^ Department of Medicine and Therapeutics, The Chinese University of Hong Kong, Hong Kong, Hong Kong SAR, China; ^6^ Department of Anaesthesia and Intensive Care, The Chinese University of Hong Kong, Hong Kong, Hong Kong SAR, China; ^7^ Institute of Chinese Medicine and State Key Laboratory of Research on Bioactivities and Clinical Applications of Medicinal Plants, The Chinese University of Hong Kong, Hong Kong, Hong Kong SAR, China; ^8^ Li Dak Sum Yip Yio Chin R & D Centre for Chinese Medicine, The Chinese University of Hong Kong, Hong Kong, Hong Kong SAR, China

**Keywords:** biomarker, COVID-19, cytokines, METRNβ, prognostic

## Abstract

**Introduction:**

Coronavirus disease 2019 (COVID-19) is increasing worldwide, with complications due to frequent viral mutations, an intricate pathophysiology, and variable host immune responses. Biomarkers with predictive and prognostic value are crucial but lacking.

**Methods:**

Serum samples from authentic and D614G variant (non-Omicron), and Omicron-SARS-CoV-2 infected patients were collected for METRNβ detection and longitudinal cytokine/chemokine analysis. Correlation analyses were performed to compare the relationships between serum METRNβ levels and cytokines/chemokines, laboratory parameters, and disease severity. Receiver operating characteristic (ROC) curves and Kaplan-Meier survival curves were used to evaluate the predictive value of METRNβ in COVID-19.

**Results:**

The serum level of METRNβ was highly elevated in non-Omicron-SARS-CoV-2 infected patients compared to healthy individuals, and the non-survivor displayed higher METRNβ levels than survivors among the critical ones. METRNβ concentration showed positive correlation with viral load in NAPS. ROC curve showed that a baseline METRNβ level of 1886.89 pg/ml distinguished COVID-19 patients from non-infected individuals with an AUC of 0.830. Longitudinal analysis of cytokine/chemokine profiles revealed a positive correlation between METRNβ and pro-inflammatory cytokines such as IL6, and an inverse correlation with soluble CD40L (sCD40L). Higher METRNβ was associated with increased mortality. These findings were validated in a second and third cohort of COVID-19 patients identified in a subsequent wave.

**Discussion:**

Our study uncovered the precise role of METRNβ in predicting the severity of COVID-19, thus providing a scientific basis for further evaluation of the role of METRNβ in triage therapeutic strategies.

## Introduction

The outbreak of severe acute respiratory syndrome coronavirus 2 (SARS-CoV-2) infection has resulted in a pandemic worldwide since the end of 2019. The number of patients suffering from Coronavirus-disease 2019 (COVID-19) has substantially increased with the rapid mutation of SARS-CoV-2 variants. The clinical manifestations of COVID-19 are heterogeneous, with the majority of patients exhibiting asymptotic to mild/moderate symptoms such as cough, sneeze, myalgia, or fatigue; however, some patients may experience systemic deterioration and progression into fatal outcomes such as pneumonia, acute respiratory distress syndrome (ARDS), coagulation disorders, and septic-related multiple organ dysfunction (MODS) (1, 2). The distinct immune response of patients to infections renders it difficult to predict early disease progression and severity.

Recent studies suggest that uncontrolled hyperinflammation (pathogenic inflammation) contributes to disease progression and severity. The death of some COVID-19 patients is strongly related to cytokine release syndrome (CRS), this is similar to septic patients characterized by excessive IL6, TNFA, IL1B, and CXCL8 levels in their blood (3–6). In addition, our previous longitudinal analysis of cytokine profiles in mild to critical COVID-19 patients revealed that cytokines such as IL10, CXCL9, CXCL10, and IL6 progressively increased with greater disease severity (7). Therefore, immunomodulatory agents such as monoclonal antibodies targeting cytokines are routinely approved for clinical trials to mitigate this SARS-CoV-2-induced pathogenic inflammation. For example, several single-center investigations have used IL6 inhibitors to treat patients with COVID-19 and demonstrated certain clinical benefits (8). In addition, immunosuppressive IL38 is a potential therapeutic biologic that downregulates inflammation in COVID-19 and provides a new anti-inflammatory treatment strategy (9, 10). However, the side effects of these drugs prompt the urgent identification of biomarkers that can help accurately predict patient outcomes and guide immunomodulatory therapeutic strategies. Recently, elevated clinical and laboratory markers (lactate dehydrogenase (LDH) (11), C-reactive protein (CRP) (12), and D-dimer (13)) were evaluated during disease progression to predict patients that are likely to have severe/critical conditions to guide treatment strategies. However, these parameters lack sensitivity and specificity. Therefore, further evaluation of the pathogenesis of SARS-CoV-2 is needed to identify novel prognostic biomarkers of COVID-19.

This study focused on METRNβ, a novel immunosuppressive cytokine/myokine/adipokine mainly secreted by activated macrophages and barrier tissues like mucosa, skin, and adipose tissue (14–16). METRNβ has strong inhibitory potential in metabolic disease, cardiac vascular diseases, and infectious diseases (mainly *via* downregulating inflammation and related pathways) (17–19). Our recent study demonstrated that METRNβ is rapidly secreted by bronchial epithelial cells and immune cells (including macrophages and eosinophils) in response to allergic pulmonary inflammation for the suppression of Th1 and Th2-mediated inflammation (20). Therefore, we propose METRNβ as a novel broad anti-inflammatory protein. However, the regulatory effects of METRNβ in COVID-19 is unknown.

We investigated whether METRNβ can help distinguish infected COVID-19 patients from healthy individuals and predict disease outcomes in the early phase of hospitalization. Therefore, we enrolled authentic and D614G variant of SARS-CoV-2 infected individuals. We further validated the findings using Omicron-SARS-CoV-2 infected individuals.

## Materials and methods

### Study design and ethics

The study enrolled adult COVID-19 patients (≥18 years of age) admitted to the Prince of Wales Hospital, Hong Kong, from February 2020 to May 2022. COVID-19 diagnosis was confirmed by positive SARSCoV-2 real time reverse transcription-polymerase chain reaction (RT-PCR) results in respiratory specimens. Briefly, viral RNA was extracted from deep throat saliva samples using the QIAamp Viral RNA Mini Kit (Qiagen, Hilden, Germany) according to the manufacturer’s instructions. The primer-probe set (F: 5’-GAC CCC AAA ATC AGC GAA AT-3’, R: 5’-TCT GGT TAC TGC CAG TTG AAT CTG-3’ and P: 5’-FAM-ACC CCG CAT TAC GTT TGG TGG ACC-BHQ1-3’) was designed by US Centers for Disease Control and Prevention (CDC) (21) and purchased from Integrated DNA Technologies, USA. The one-step real-time RT-PCR reaction contained 5 μL of the extracted preparation, 4 μL TaqMan™ Fast Virus 1-Step Master Mix (Applied Biosystems, Waltham, MA, USA) in a final reaction volume of 20 μL. The primer and probe concentration was 0.5 μM and 0.125 μM, respectively. The cycling conditions, 25°C for 2 min, 50°C for 15 min, 95°C for 2 min, followed by 45 cycles of 95°C for 15 s, and 55°C for 30 s, were performed with the StepOnePlus Real-Time PCR System (Applied Biosystems, USA). The cycle threshold (Ct) values of real time RT-PCR were converted into viral RNA copies based on a standard curve prepared from 10-fold serial dilutions of know copies of plasmid containing the full N gene (2019-nCoV_N_Positive Control, Integrated DNA Technologies, Coralville, IA, USA). Samples were considered as negative if the Ct values exceeded 39.9 cycles. The detection limit of real-time RT PCR was 694 copies/mL. The exclusion criteria included bone marrow aplasia, pregnancy, breastfeeding, HIV (human immunodeficiency virus) infection with low CD4 T-cell count (< 200/mL), or patients receiving immunosuppressive therapy. COVID-19 disease severity was classified as we previously described (7). Serum samples were collected within several days of admission and another set of samples was collected at 4, 7, and 10 days after admission, depending on feasibility. In addition, convalescent samples were obtained from some patients. The COVID-19 treatment strategy in our hospital has evolved over time, including the application of interferon, lopinavir-ritonavir, ribavirin, remdesivir, tocilizumab, oxygen inhalation, and mechanical ventilation. Eligible participants provided written informed consent according to the Declaration of Helsinki. Written informed consent was provided by the surrogate decision maker and later confirmed by the patients themselves as required during ICU management. The study was approved by the Chinese University of Hong Kong New Territories East Cluster Clinical Research Ethics Committee.

### Quantification of serum METRNβ levels

Venous blood was collected soon after hospitalization, and the serum was separated by centrifuging the blood at 1000g for 10 min at 4°C and stored at –80°C until further use. METRNβ concentration was measured using enzyme-linked immunosorbent assay (ELISA) (R&D Systems Inc., Minneapolis, MN, USA) according to the Manufacturer’s instructions. Briefly, a plate was coated with 100 μL diluted capture antibody overnight at room temperature. After washing 3 times with wash buffer, the plate was blocked with 1% BAS in PBS for 2h at room temperature, followed by incubating with 100 μL diluted samples/standards for 2h at room temperature, 100 μL detection antibody for 2 hours at room temperature, 100 μL working dilution of Streptavidin-HRP for 20 minutes at room temperature in dark, and 100 μL of substrate solution for 20 minutes at room temperature in dark. Repeat the wash procedure after each step. After the last wash, the reaction was stopped by incubating with 50 μL stop solution and the optical density immediately was determined using a microplate reader set to 450 nm. We found that age factor has no significant influence on the METRNβ levels, while the concentrations of METRNβ among different age groups were not significant different (p>0.05).

### Longitudinal cytokine profile

Serum concentrations of human soluble CD40 ligand (sCD40L), epidermal growth factor (EGF), fibroblast growth factor 2 (FGF2), FMS-like tyrosine kinase 3 ligand (FLT3L), C-X3-C motif chemokine ligand 1(CX3CL1), colony stimulating factor 3(CSF3), granulocyte macrophage colony-stimulating factor (GMCSF), Interferon alpha-2 (IFNA2), interferon gamma (IFNG), interleukin (IL)1A, IL1B, IL1R1, IL2, IL3, IL4, IL5, IL6, IL7, IL8/CXCL8, IL9, IL10, IL12p40, IL12p70, IL13, IL15, IL17A, IL18, C-C motif chemokine ligand (CCL)2, CCL3, CCL4, CCL7, CCL11, CCL22, C-X-C motif chemokine ligand (CXCL)1, CXCL9, CXCL10, transforming growth factor alpha (TGFA), tumor necrosis factor alpha (TNFA), tumor necrosis factor beta (TNFB), and vascular endothelial growth factor A (VEGFA) were quantified using a human Cytokine Milliplex MAP assay kit (Millipore Corporation, Billerica, MA, USA) using a Bio-Plex 200 system (Bio–Rad Laboratories, Hercules, CA, USA) according the manufacturer’s instructions.

### Measurement of biochemical parameters

Serum levels of C-reactive protein (CRP), lactate dehydrogenase (LDH), alkaline phosphatase (ALP), creatine kinase (CK), D-dimer, and urea were assessed by the routine chemical pathology service laboratory, Prince of Wales Hospital, Shatin, Hong Kong.

### Statistical analysis

All statistical analyses were performed using GraphPad Prism software (version 9.0; La Jolla, CA, USA) and IBM SPSS (version 26.0; Armonk, New York, USA). Because of non-Gaussian distribution of data, the parameters between the groups were compared using the nonparametric Mann–Whitney U test, Wilcoson test or Kruskal–Wallis test followed by Dunn’s multiple comparisons post-test as appropriate. Non-parametric Spearman’s rank correlation test was used to evaluate the correlation of METRNβ concentrations with multiple cytokines/chemokines concentrations together with laboratory parameters. Receiver operating characteristic (ROC) curves were constructed and the area under the curve (AUC) was determined to evaluate the predictive value of METRNβ. The optical cutoff of METRNβ levels was confirmed based on the maximized weighted combination of sensitivity and specificity (Youden index). Kaplan–Meier plots and log-rank tests were conducted to analyze the differences in survival probabilities based on the cutoff points after patients were stratified between the low and high levels of METRNβ across the follow-up timeframe. This was calculated from the date of cytokine testing to the date of death, discharge, or end of the follow-up period as appropriate. Significance was set at *P < 0.05, **P < 0.01, and ***P < 0.001.

## Results

### Patient recruitment and characteristics

A total of 302 patients and 182 healthy donors (without any acquired, baseline drug-induced, or congenital immunosuppression) were included. Analysis involved dividing patients into three different groups: (1) prediction cohort infected with authentic and D614G variant of SARS-CoV-2 (non-Omicron-SARS-CoV-2) (Supplementary Table 1); (2) validation cohort infected with non-Omicron-SARS-CoV-2 (Supplementary Table 2); and (3) validation cohort of patients infected with Omicron SARS-CoV-2 (Supplementary Table 3).

### METRNβ levels in the prediction cohort of non-Omicron-SARS-CoV-2 infected COVID-19 patients

**Figure 1:**
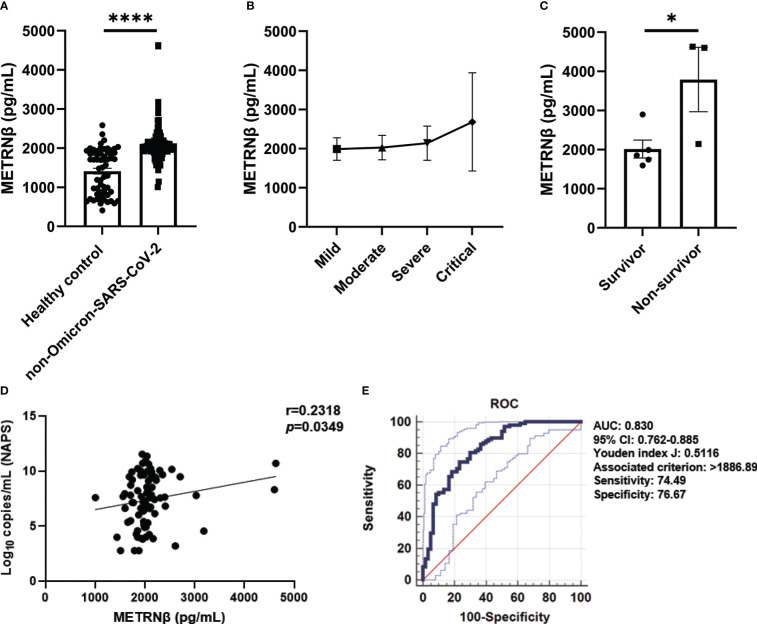
METRNβ expression increased in non-Omicron-SARS-CoV-2 infected COVID-19 patients of the prediction cohort. **(A)** Circulating METRNβ concentrations in COVID-19 patients (n = 98) and healthy controls (n = 60) detected by ELISA. **(B)** COVID-19 patients were stratified into mild (n = 36), moderate (n = 41), severe (n = 13), and critical groups (n = 8), and circulating METRNβ levels in serum were detected by ELISA. **(C)** Serum METRNβ concentration of the non-survivors (n = 3) and survivors (n = 5) among the critical patients was measured by ELISA. **(D)** Correlation analysis of serum METRNβ concentrations with viral loads in the NAPS in patients with non-Omicron-SARS-CoV-2 infections (n = 83). **(E)** Receiver operating characteristic curves of serum METRNβ levels on admission to distinguish COVID-19 patients from healthy controls. The AUC was 0.830 (95% CI: 0.762–0.885) for serum METRNβ levels on admission in the prediction cohort of COVID-19 patients with non-Omicron-SARS-CoV-2 infection (n = 98). Data were shown as the mean ± SEM. The Mann–Whitney test was used to compare the differences between groups. Spearman’s correlation coefficient was used in the statistics for correlation analysis. NAPS, nasopharyngeal swab specimens; AUC, area under the curve. *P < 0.05 and ****P < 0.0001.

METRNβ levels significantly increased (1,004–4,630 pg/mL) in the serum of ninety-eight authentic and D614G variant of SARS-CoV-2 (non-Omicron-SARS-CoV-2) infected COVID-19 patients collected soon after admission compared to healthy controls (412.3–2,584 pg/mL) (**Figure 1A**). A gradual increase in METRNβ levels was detected in critical cases (1,595–4,630 pg/mL) compared to mild cases (1,144–2,708 pg/mL) after dividing the patients into mild, moderate, severe, and critical subgroups based on their clinical outcomes (**Figure 1B**). Notably, in the critical groups, non-survivors displayed significantly higher METRNβ concentrations at the time of admission than those who survived (**Figure 1C**). The correlation between METRNβ and clinical parameters [including CRP, LDH, ALP, D-dimer, and viral load in nasopharyngeal swab specimens (NAPS)] was compared. There was a significant positive correlation between METRNβ levels and viral load in NAPS (*p*=0.0349) (**Figure 1D**), whereas METRNβ had a slightly positive correlation with infected patient CRP, LDH, and ALP concentrations (**Figures S1A–C**).

We propose that serum METRNβ may be a surrogate indicator of COVID-19 based on the substantial differences in serum METRNβ levels between non-Omicron-SARS-CoV-2 infected patients and healthy controls. Therefore, ROC curves were prepared to calculate the best cut-off value to distinguish SARS-CoV-2 infected individuals from healthy controls. The AUC was 0.830 (95% CI: 0.762–0.885), and the optimal cut-off for METRNβ to distinguish SARS-CoV-2 infected individuals from healthy controls was 1886.89 pg/mL, with sensitivity 74.49% and specificity 76.67% (**Figure 1E**). Together, the results reveal a marked increase in METRNβ in COVID-19 patients and its levels correlated with disease severity.

### Correlation of METRNβ with cytokine storm in non-Omicron-SARS-CoV-2 infected patients

**Figure 2:**
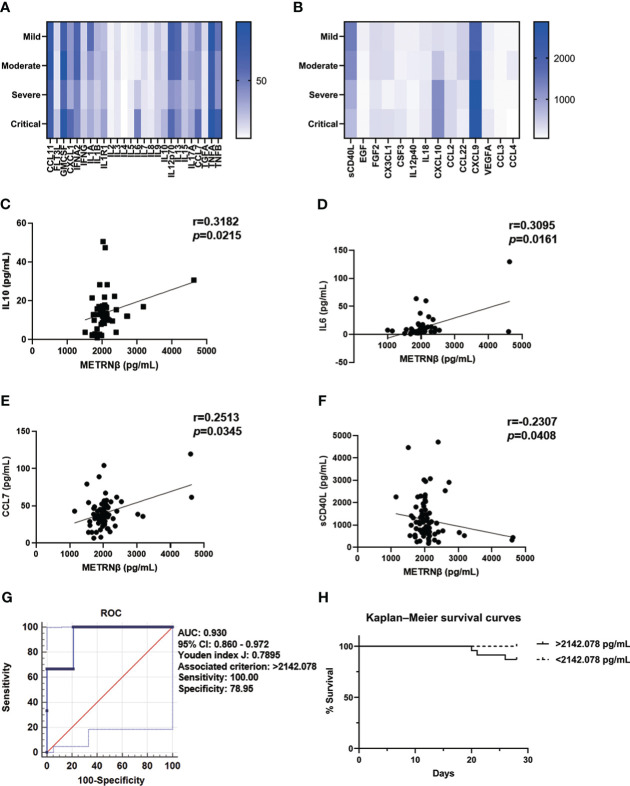
METRNβ served as a surrogate prognostic biomarker in COVID-19 patients in the prediction cohort. **(A, B)** Longitudinal cytokine/chemokine profile of COVID-19 patients (n = 54–98) with the human Cytokine Milliplex MAP assay kit. The results are shown as heat maps. **(C–F)** Correlation analysis of serum METRNβ concentration with IL10 (n = 52), IL6 (n = 60), CCL7 (n = 71), and sCD40L (n = 79). Spearman’s correlation coefficient was used for the analysis. **(G)** ROC of serum METRNβ levels on admission to distinguishing survivors from non-survivors of COVID-19 patients. The AUC was 0.930 (95% CI: 0.860–0.972) for serum METRNβ levels on admission in the prediction cohort of COVID-19 patients with non-Omicron-SARS-CoV-2 infection (n = 98). **(H)** Kaplan–Meier survival curves of COVID-19 patients with non-Omicron-SARS-CoV-2 infections in the prediction cohort based on the METRNβ cut-off value (2142.078 pg/mL) on the day of admission. AUC, area under the curve.

Cytokine release syndrome contributes to multiple organ failure in COVID-19 patients and results in fatal outcomes (22). The “cytokine release syndrome” has been used to clinically define higher mortality risk in COVID-19 patients (23). Therefore, we further used a rapid multiplex immunoassay to measure the longitudinal change in cytokine/chemokine profiles of COVID-19 patients after they were stratified by disease severity. There was an elevation of a wide range of cytokines/chemokines, including CXCL1, IL6, IL10, CCL7, TNFB, CXCL9, and CXCL10, while lowered cytokine levels such as sCD40L in the severe/critical cases compared with the mild/moderate cases (**Figures 2A, B**). Next, correlation analysis was performed between METRNβ and these cytokines/chemokines to reveal the possible relationship of METRNβ with the disturbed immune response of COVID-19 patients. A positive correlation of METRNβ with IL10 (**Figure 2C**), IL6 (**Figure 2D**), and CCL7 (**Figure 2E**) was observed among the COVID-19 patients, while a negative correlation was observed with sCD40L (**Figure 2F**). Overall, these results support the positive correlation between METRNβ, disease severity, and pathogenic inflammation in COVID-19 patients.

### Predictive and prognostic value of METRNβ in non-Omicron-SARS-CoV-2 infected patients

We propose that serum METRNβ may be useful in predicting COVID-19 outcome based on the substantial higher METRNβ concentrations in the severe/critical COVID-19 patients than mild/moderate patients. Therefore, ROC curves were prepared to calculate the best cut-off value of METRNβ to predict SARS-CoV-2 infection associated mortality. The AUC was 0.930 (95% CI: 0.860–0.972) for METRNβ to distinguish non-survivors from survivors (**Figure 2G**), with the calculated Youden index (0.7895) and the associated METRNβ cut-off point (2142.078 pg/mL). This value had a sensitivity of 100% and specificity of 78.95%. Next, Kaplan–Meier survival curves were established among the COVID-19 patients after they were stratified according to serum METRNβ levels on the day of hospital admission, with a cutoff point of 2142.078 pg/mL. Patients with higher serum METRNβ levels (>2142.078 pg/mL) had worse survival than those with lower METRNβ concentrations (**Figure 2H**). The results indicate that the early detection of METRNβ may help in the prediction the outcome of SARS-CoV-2 infected COVID-19 patients.

### Validation of METRNβ as a predictive biomarker in non-Omicron-SARS-CoV-2 infected patients

**Figure 3:**
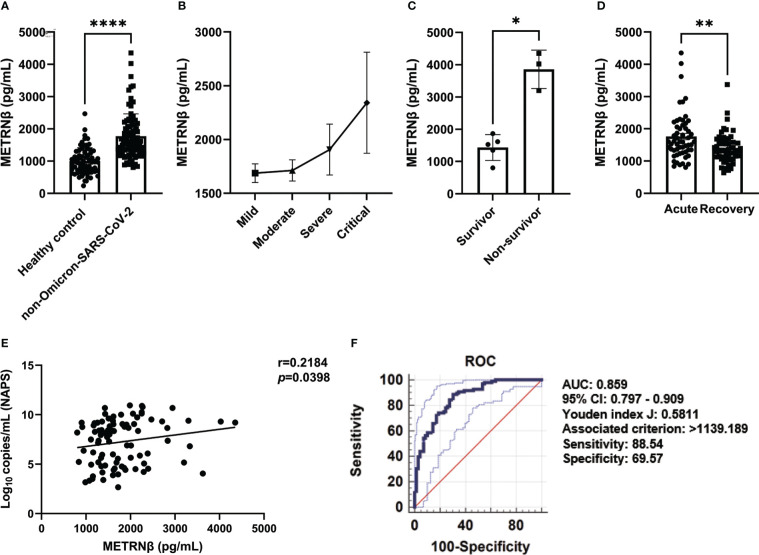
METRNβ concentration substantially increased in the non-Omicron-SARS-CoV-2 infected COVID-19 patients in the validation cohort. **(A)** Circulating METRNβ concentrations in COVID-19 patients (n = 96) and healthy controls (n = 69) detected by ELISA. **(B)** COVID-19 patients were stratified into mild (n = 41), moderate (n = 38), severe (n = 9), and critical groups (n = 8), and circulating METRNβ levels in serum detected by ELISA. **(C)** Serum METRNβ concentration of the non-survivors (n = 3) and survivors (n = 5) in the critical group measured by ELISA. **(D)** Circulating METRNβ concentrations in COVID-19 patients in the acute and recovery phase (pre and post-treatment) measured by ELISA (n = 56). **(E)** Correlation analysis of serum METRNβ concentration with viral loads in the NAPS of patients with non-Omicron-SARS-CoV-2 infections (n = 89). **(F)** ROC of serum METRNβ levels to distinguish COVID-19 patients from healthy controls. The AUC was 0.859 (95% CI: 0.797–0.909) for serum METRNβ levels on admission in the validation cohort of COVID-19 patients with non-Omicron-SARS-CoV-2 infection (n = 96). Data were shown as the mean ± SEM. Student’s paired t test **(D)** and/or Mann–Whitney test, or the Kruskal–Wallis test followed by Dunn’s multiple comparisons post-test was used to compare the differences between groups where appropriate. Spearman’s correlation coefficient was used for correlation analysis. NAPS, nasopharyngeal swab specimens; AUC, area under the curve. *P < 0.05, **P < 0.01, and ****P < 0.0001.

Another ninety-six authentic and D614G variant of SARS-CoV-2 infected patients were recruited as our validation cohort. METRNβ levels were markedly higher in infected individuals than in healthy controls (**Figure 3A**). Severe/critical patients displayed increased METRNβ levels compared with the mild/moderate patients (**Figure 3B**). Importantly, the fatal cases in the critical groups of validation cohort also showed higher METRNβ levels on the day of administration compared to those who survived (**Figure 3C**). METRNβ levels returned to normal when patients recovered from the acute phase (**Figure 3D**). Furthermore, METRNβ levels positively correlated with viral load in NAPS (*p*=0.0398) (**Figure 3E**). ROC analysis revealed an AUC of 0.859 for METRNβ to identify COVID-19 patients from healthy controls, and a cut-off of METRNβ at 1139.189 pg/mL can effectively indicate COVID1-9 infections with sensitivity (88.54%) and specificity (69.57%) (**Figure 3F**).

**Figure 4:**
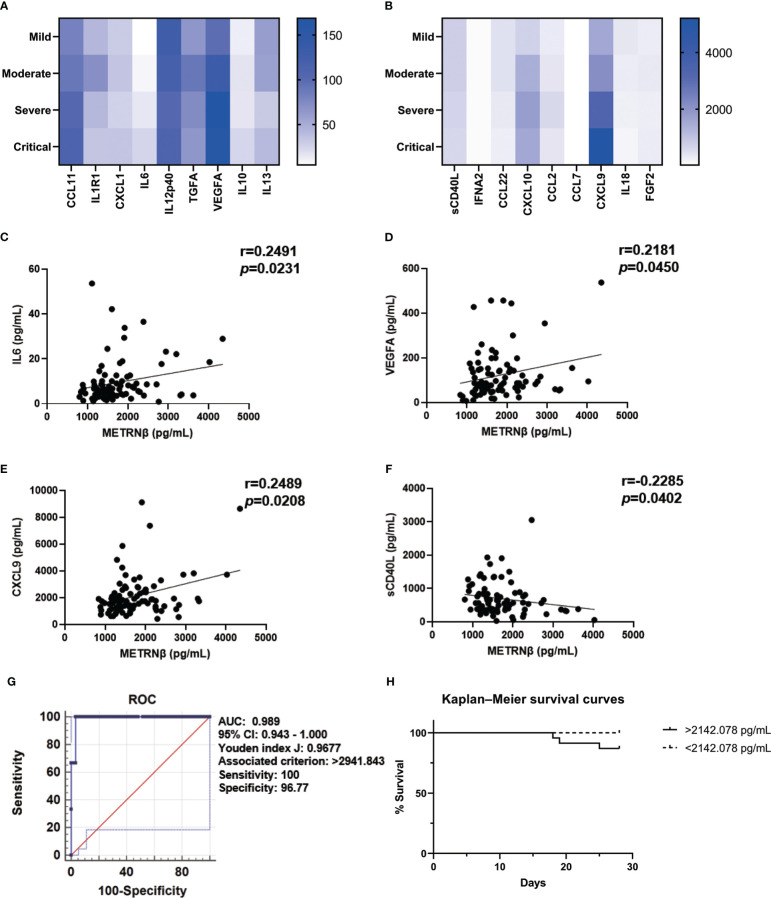
Validation of METRNβ as a surrogate prognostic biomarker for COVID-19. **(A, B)** Longitudinal cytokine/chemokine profile of COVID-19 patients (n = 81–96) with the human Cytokine Milliplex MAP assay kit. The results were shown as heat maps. **(C–F)** Correlation analysis of serum METRNβ concentration with IL6 (n = 83), VEGFA (n = 85), CXCL9 (n = 86) and sCD40L (n = 81). Spearman’s correlation coefficient was used for correlation analysis. **(G)** ROC of serum METRNβ at admission to distinguish non-survivors from survivors. The AUC was 0.989 (95% CI: 0.943–1.000) for serum METRNβ levels on admission in the validation cohort of COVID-19 patients with non-Omicron-SARS-CoV-2 infections (n = 96). **(H)** Kaplan-Meier survival curves of COVID-19 patients with non-Omicron-SARS-CoV-2 infections in the validation cohort based on the METRNβ cutoff value (2142.078 pg/mL) calculated from the prediction cohort. AUC: area under the curve.

Notably, severe/critical patients in the validation cohort also displayed a greater pro-inflammatory status with higher concentrations of CCL11, IL6, VEGFA, IL10, CXCL10, and CXCL9, compared to the mild/moderate ones (**Figures 4A, B**). Circulating METRNβ concentrations positively correlated with IL6, VEGFA, and CXCL9 (**Figures 4C–E**), and inversely correlated with sCD40L (**Figure 4F**). These results were consistent with the prediction cohort and provided further evidence that higher METRNβ concentrations are associated with a more severe disease phenotype. Furthermore, receiver operator characteristic analysis determined an AUC of 0.989 (95% CI: 0.942–1.000) for METRNβ to distinguish non-survivors with survivors among the COVID-19 patients (**Figure 4G**). Kaplan–Meier survival curves for patients in the validation cohort were established with a 2142.078 pg/mL METRNβ cut-off value calculated from the prediction cohort to investigate the prediction value of METRNβ in COVID-19 outcomes. Patients with high METRNβ (> 2142.078 pg/mL) were associated with an increased mortality of COVID-19 (**Figure 4H**). Together, these results validate that METRNβ may be a prognostic marker of COVID-19 severity and death.

### Validation of METRNβ as a predictive biomarker in Omicron-SARS-CoV-2 infected patients

**Figure 5:**
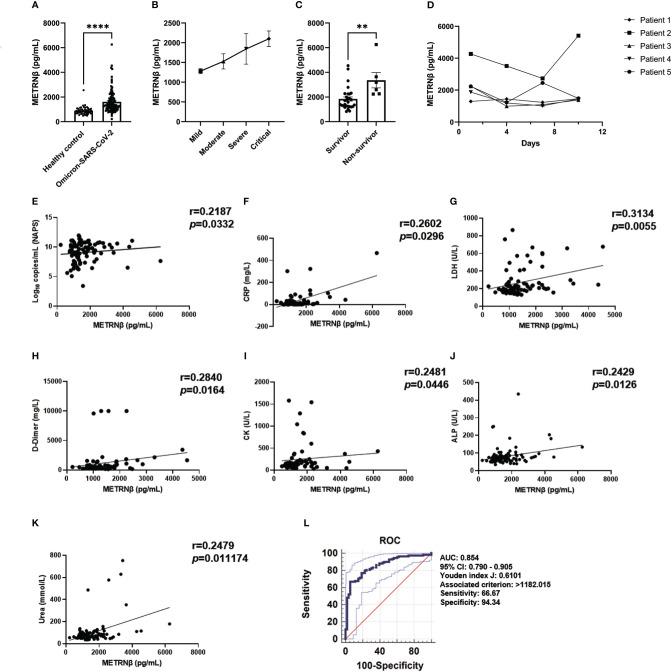
METRNβ concentration was elevated in the validation cohort of Omicron-SARS-CoV-2 infected COVID-19 patients on admission. **(A)** Circulating METRNβ concentrations in COVID-19 patients infected with Omicron-SARS-CoV-2 (n = 108) and healthy controls (n = 53) detected by ELISA. **(B)** COVID-19 patients were stratified into mild (n = 54), moderate (n = 9), severe (n = 10), and critical groups (n = 35). Circulating METRNβ levels in serum were detected with ELISA. **(C)** Serum METRNβ concentration of the non-survivors (n = 6) and survivors (n = 29) among the critical patients measured by ELISA. **(D)** Dynamic change of circulating METRNβ concentrations in 5 Omicron-COVID-19 patients on the day of administration and 4 days, 7 days, and 10 days after administration (n = 5). **(E)** Correlation analysis of serum METRNβ concentration with viral loads in the NAPS in patients with Omicron SARS-CoV-2 infections (n = 95). **(F–K)** Correlation analysis of serum METRNβ concentration with CRP (n = 70), LDH (n = 77), D-dimer (n = 71), CK (n = 66), ALP (n = 105), and urea (n = 104) in patients with Omicron-SARS-CoV-2 infections. **(L)** ROC of serum METRNβ levels on admission to distinguish COVID-19 patients from healthy controls. The AUC was 0.854 (95% CI: 0.790–0.905) for serum METRNβ levels on admission in the validation cohort of Omicron-SARS-CoV-2 infections (n = 108). Data were shown as the mean ± SEM. The Mann–Whitney test or Kruskal–Wallis test followed by Dunn’s multiple comparisons post-test was used to compare the differences between groups as appropriate. Spearman’s correlation coefficient was used for correlation analysis. NAPS: nasopharyngeal swab specimens. CRP, C-reactive protein; LDH, lactate dehydrogenase; ALP, alkaline phosphatase; CK, creatine kinase; AUC, area under the curve. **P < 0.01 and ****P < 0.0001.

Several SARS-CoV-2 subtypes have recently appeared, which display various symptoms and outcomes upon infection owing to the rapid mutation of the virus. Therefore, one hundred and eight omicron-SARS-CoV-2 infected individuals were additionally recruited to address whether the observed predictive value of METRNβ in non-Omicron-SARS-CoV-2 may apply to other variants (Supplementary Table 3). METRNβ substantially increased in Omicron-SARS-CoV-2-infected individuals compared with healthy individuals (**Figure 5A**) and severe cases displayed higher METRNβ levels than mild/moderate cases (**Figure 5B**). These results were similar to the prediction and validation cohorts of non-Omicron-SARS-CoV-2-infected individuals. Of note, patients who died during the study period displayed substantially higher METRNβ levels than those who survived among the critical patients (**Figure 5C**). The dynamic change in METRNβ levels along with disease progression were also monitored for five patients without treatment during the study period. Although there was a slight decline in METRNβ in one patient on day 7, METRNβ levels remained steady at high concentrations before their recovery (**Figure 5D**). This indicated that METRNβ was maintained at a stable elevated level. METRNβ concentrations showed significant positive correlation with NAPS viral load (*p*=0.0332) (**Figure 5E**), and laboratory parameters that may reflect organ damage and disturbance of the host, including CRP (*p*=0.0296), LDH (*p*=0.0055), D-dimer (*p*=0.0164), CK (*p*=0.0446), ALP (*p*=0.0126), and urea (*p*=0.011174) in Omicron-SARS-CoV-2 infected individuals (**Figures 5F–K**). Notably, ROC analysis calculated a cutoff of METRNβ level at 1182.015 pg/mL can effectively distinguish Omicron COVID-19 patients from healthy controls, with sensitivity 66.67%, and specificity 94.34%. The AUC was 0.854 (**Figure 5L**). Overall, these results confirmed that METRNβ may also distinguish infected patients from non-infected healthy controls in the Omicron-SARS-Cov-2 infected study cohort.

**Figure 6:**
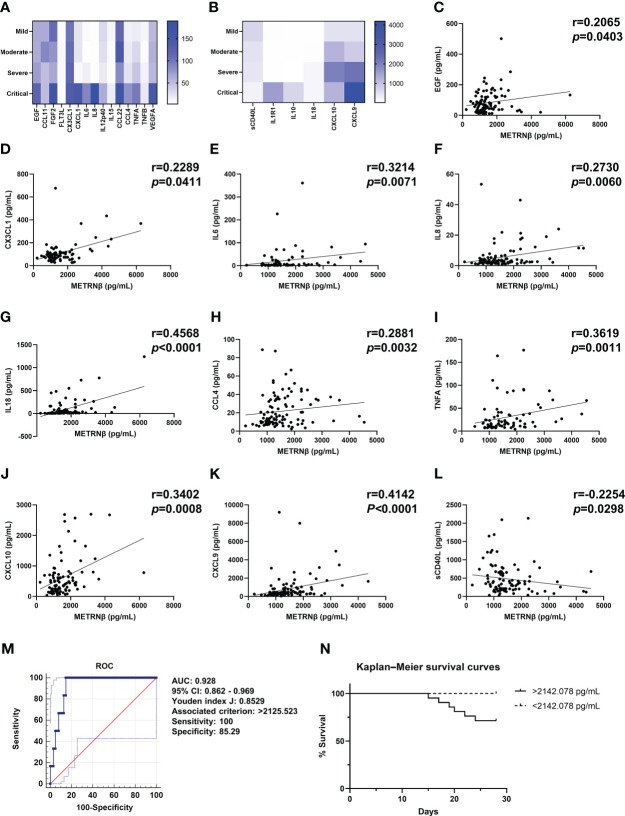
Validation of METRNβ as a biomarker in patients infected with Omicron-SARS-CoV-2. **(A, B)** Longitudinal cytokine/chemokine profile of COVID-19 patients with Omicron-SARS-CoV-2 infections (n = 69–108) analyzed using the human Cytokine Milliplex MAP assay kit. The results were shown as heat maps. **(C–L)** Correlation analysis of serum METRNβ concentration with EGF (n = 99), CX3CL1 (n = 80), IL6 (n = 69), IL8 (n = 100), IL18 (n = 102), CCL4 (n = 103), TNFA (n = 78), CXCL10 (n = 93), CXCL9 (n = 101), and sCD40L (n = 93). Spearman’s correlation coefficient was used for correlation analysis. **(M)** ROC of serum METRNβ at admission to distinguish non-survivors from survivors among the Omicron-COVID-19 patients. The AUC was 0.928 (95% CI: 0.862–0.969) for serum METRNβ levels at admission in the validation cohort of COVID-19 patients with Omicron-SARS-CoV2 infections (n = 108). **(N)** Kaplan–Meier survival curves of COVID-19 patients in the Omicron validation cohort based on the METRNβ cutoff value (2142.078 pg/mL) calculated from non-omicron prediction cohort. AUC, area under the curve.

Longitudinal cytokine/chemokine profile analysis revealed more pro-inflammatory cytokine/chemokine maps in severe/critical Omicron-SARS-CoV-2 infected individuals compared with that for mild/moderate patients. This is highlighted by increased levels of EGF, FGF2, CX3CL1, CXCL1, IL6, IL8, IL-12p40, CCL4, TNFA, VEGFA, IL1R1, IL10, IL18, CXCL10, and CXCL9 (**Figures 6A, B**), while there was less sCD40L in severe/critical cases. The results are similar to those of the prediction and validation cohorts infected with non-Omicron-SARS-CoV-2. METRNβ concentrations also displayed positive correlations with EGF, CX3CL1, IL6, IL8, IL18, CCL4 TNFA, CXCL10, and CXCL9 (**Figures 6C–K**), and an inverse correlation with sCD40L (**Figure 6L**). Together, these results provide evidence that METRNβ concentrations correlate with disease severity in the Omicron-SARS-CoV-2 infected groups on the day of administration. Finally, an AUC of 0.928 (95% CI: 0.862–0.969) was identified for METRNβ in identifying non-survivors from survivors among the Omicron-SARS-CoV-2 infected patient using ROC (**Figure 6M**). Based on the cutoff value of METRNβ at 2142.078 pg/mL calculated from the non-Omicron prediction cohort, we further stratified patients into METRNβ > 2142.078 pg/mL groups and METRNβ < 2142.078 pg/mL groups on their administration and performed Kaplan–Meier analysis thereafter. Patients with higher METRNβ concentrations had a higher probability of death (**Figure 6N**). These results validate that METRNβ may also be a prognostic marker in of Omicron-SARS-CoV-2 infection related death.

## Discussion

There are conflicting findings of SARS-CoV-2 infection prognostic markers (24). Some laboratory markers such as CRP and procalcitonin (PCT) increase in COVID-19 infection (25). however, these markers cannot predict patient outcomes and lack specificity (26, 27). The disturbed immune response characterized by the cytokine storm strongly contributes to the COVID-19-related fatality (28–30). Therefore, early cytokine measurements become reliable predictors of disease severity and outcomes and is critical to guide treatment decision. METRNβ has been considered as a cytokine, and its levels are reported to be elevated in inflammation (17, 31), therefore, METRNβ may be a potential candidate for monitor of COVID-19 related progress and outcome. This study found that METRNβ might serve as a surrogate marker for the identification and prognosis of COVID-19 severity in both the prediction and validation cohorts of patients infected with non-Omicron-SARS-CoV2 and Omicron SARS-CoV-2. This may help in patient stratification and provide an early target clinical therapy.

Both authentic, D614G variant and Omicron SARS-CoV-2 infections led to a sustained and stable increase in circulating METRNβ concentration, and the severe/critical cases displayed higher METRNβ levels than the mild/moderate cases. The positive correlation of elevated METRNβ levels with disease severity parameters such as D-dimer (32), CRP, LDH (33) and ALP (34), and that elevated METRNβ levels in parallel with the longitudinal elevation of multiple cytokines/chemokines for the pathogenic cytokine storm of COVID-19, show that METRNβ is a valuable prognostic marker of COVID-19. As METRNβ is considered a cytokine (31, 35, 36), therefore, these findings also suggest that METRNβ contributes to the modulation of the cytokine storm in COVID-19.

A longitudinal increase in a variety of inflammatory cytokines, including IL6, IL8/CXCL8, and TNFA, in COVID-19 patients has been observed (22, 37, 38). However, very few studies have examined their prognostic value. Furthermore, they lack specificity since they are known markers of inflammation and organ damage (39, 40). In addition, many cytokines exhibit relatively low concentrations (mostly at pg/mL); therefore, quantitation requires relatively large volumes of clinical samples to ensure sensitivity and specificity (41, 42). Our study showed that METRNβ is a superior biomarker of COVID-19, because it sustains a stable high concentration in the circulation to allow effective monitoring. More importantly, METRNβ levels were upregulated within several days of admission, making it a useful biomarker in the early prediction of possible severe outcomes of COVID-19. Early stratification of severe or critical patients using the METRNβ assay will be beneficial for the optimization of hospital resources in the new area of personalized therapy. The measurement of METRNβ has one additional advantage. The biomarker potential of METRNβ was validated in patients infected with several distinct clades of viruses, therefore, METRNβ may be applied to the identification and prediction of general SARS-CoV-2 infections.

However, we also acknowledge some limitations. First, our sample size and type are limited. Larger sample size including more SARS-CoV-2 clade like Delta would provide more evidence of characterizing METRNβ as a COVID-19 prognostic marker. Second, understanding the function of the METRNβ protein is still lacking, further functional studies should warrant to determine if METRNβ is safe to target or if METRNβ plays a different physiological role for homeostasis in patients. Finally, since the METRNβ levels positively correlated with the viral load of the patients, METRNβ might be able to suppress the antiviral capabilities of the host. Although suitable METRNβ concentrations may be able to suppress the immunopathological injury mediated by excessive inflammatory reaction because of its anti-inflammatory capabilities, excessive and sustained elevation of METRNβ may lead to immunosuppressive activity, thereby suppressing the anti-viral immunity and facilitating the viral proliferation and subsequent virus-mediated immunopathological damage, even leading to the secondary infections. Therefore, this warrants in-depth studies regarding the participation of METRNβ in the pathogenesis of COVID-19.

In conclusion, we found a significant elevation in circulating METRNβ concentrations in authentic, D614G variant and Omicron-SARS-CoV-2-infected patients. METRNβ levels were more pronounced in patients with severe or critical conditions. The robust increase in METRNβ in the disease settings and its positive correlation with disease severity make it a valuable surrogate prognostic marker of COVID-19. The predictive value of METRNβ should help guide therapeutic interventions to determine which patients are more likely to develop severe conditions, such as respiratory failure and multiple organ damage. This provides early targeted therapies to disrupt the underlying inflammation.

## Data availability statement

The original contributions presented in the study are included in the article/**Supplementary Material**. Further inquiries can be directed to the corresponding author.

## Ethics statement

The studies involving human participants were reviewed and approved by The Chinese University of Hong Kong New Territories East Cluster Clinical Research Ethics Committee. The patients/participants provided their written informed consent to participate in this study.

## Author contributions

C-KW: project administration and supervision. PC and C-KW: funding acquisition. XG and C-KW: conceptualization and formal analysis. XG, KW, AY, and DH: investigation, data curation and formal analysis. PC, RN, LL, DH, and GL: investigation, recruitment of patient samples and sorting of patient clinical information. XG and C-KW: drafted the manuscript. C-KW and PC: revised and edited the manuscript. All authors contributed to the article and approved the submitted version.
