# Supplementary material for: Characterization of METRNβ as a novel biomarker of Coronavirus disease 2019 severity and prognosis
Source: Front Immunol. 2023 Jan 31;14:1111920. doi: 10.3389/fimmu.2023.1111920 (PMC9927217; doi:10.3389/fimmu.2023.1111920)
Supplement: Supplementary file 1 [file DataSheet_1.docx]

**Supplementary materials**

**Characterization of METRNβ as a novel biomarker of Coronavirus disease 2019 severity
and prognosis**

Xun Gao^1,2^, Paul Kay-Sheung Chan^3,4^, Katie Ching-Yau Wong^2^, Rita Wai-Yin Ng^3^, Apple Chung-Man Yeung^3^, Grace Chung-Yan Lui^4,5^, Lowell Ling^6^, David Shu-Cheong Hui^4,5^, Danqi Huang^2^, and Chun-Kwok Wong^2,7,8^

^1^ Center of Clinical Laboratory Medicine, Zhongda Hospital, Southeast University, Nanjing, China; ^2^ Department of Chemical Pathology, The Chinese University of Hong Kong, Hong Kong, China; ^3^ Department of Microbiology, The Chinese University of Hong Kong, Hong Kong, China;

^4^ Stanley Ho Centre for Emerging Infectious Diseases, The Chinese University of Hong Kong, Hong Kong, China;

^5^ Department of Medicine and Therapeutics, The Chinese University of Hong Kong, Hong Kong, China;

^6^ Department of Anaesthesia and Intensive Care, The Chinese University of Hong Kong, Hong Kong, Hong Kong, China;

^7^ Institute of Chinese Medicine and State Key Laboratory of Research on Bioactivities and Clinical Applications of Medicinal Plants, The Chinese University of Hong Kong, Hong Kong, China;

^8^ Li Dak Sum Yip Yio Chin R & D Centre for Chinese Medicine, The Chinese University of Hong Kong, Hong Kong, China.

*** Correspondence:**

Professor Chun-Kwok Wong

Department of Chemical Pathology
The Chinese University of Hong Kong
Prince of Wales Hospital, Shatin, N.T., Hong Kong, China

Tel: (852) 3505 2964; Fax: (852) 2636 5090; E-Mail: [ck-wong@cuhk.edu.hk](mailto:ck-wong@cuhk.edu.hk)

**Supplemental Table 1. Demographics and characteristics of patients infected with authentic and D614G variant of SARS-CoV-2 in the prediction cohort and the corresponding healthy controls.**

| Characteristics | Mild  (N=36) | Moderate  (N=41) | Severe  (N=13) | Critical  (N=8) | Total  (N=98) | NC  (N=60) |
| --- | --- | --- | --- | --- | --- | --- |
| Sex, male/female | 6/30 | 20/21 | 7/6 | 4/4 | 37/61 | 27/33 |
| Age (years, mean±SD) | 34.7±14.5 | 46.1±15.6 | 60.7±10.7 | 60.0±15.5 | 45.1±17.0 | 41.57±14.57 |
| Complications | 0 | 0 | 5 | 8 | 13 | N/A |
| Anti-virus therapy | 8 | 15 | 13 | 8 | 44 | N/A |
| Log_10_ viral load on admission, (mean±SD in copies/mL) | 7.4±2.2 | 7.3±2.6 | 7.9±2.3 | 6.5±2.1 | 7.3±2.4 | N/A |
| Co-morbidities | 11 | 17 | 9 | 1 | 38 | N/A |
| Respiratory care (oxygen) | 0 | 0 | 10 | 8 | 18 | N/A |
| Mechanical ventilation | 0 | 0 | 0 | 2 | 2 | N/A |
| Death | 0 | 0 | 0 | 3 | 3 | N/A |

Note: Comorbidities: Coronary heart disease, hypertension, cerebrovascular, stroke, neoplastic, chronic lung, liver, and renal diseases; diabetes mellitus; autoimmune disorders; and allergic diseases.

Complications: clinico-radiographic pneumonia, bronchitis, acute exacerbation of chronic pulmonary diseases, acute cardiovascular/cerebrovascular events, and renal and metabolic derangements.

NC: sex and aged paired healthy individuals without any known medical conditions.

**Supplemental Table 2. Demographics and characteristics of patients infected with authentic and D614G variant of SARS-CoV-2 and healthy controls in the validation cohort.**

| Characteristics | Mild  (N=41) | Moderate  (N=38) | Severe  (N=9) | Critical  (N=8) | Total  (N=96) | NC  (N=69) |
| --- | --- | --- | --- | --- | --- | --- |
| Sex, male/female | 4/37 | 10/28 | 4/5 | 4/4 | 22/74 | 31/38 |
| Age (years, mean±SD) | 44.5±16.9 | 53.5±14.8 | 68.1±11.1 | 67.9±9.3 | 52.3±17.1 | 49.32±13.74 |
| Complications | 1 | 4 | 6 | 8 | 19 | N/A |
| Anti-virus therapy | 4 | 8 | 9 | 8 | 29 | N/A |
| Log_10_ viral load on admission, (mean±SD in copies/mL) | 6.7±2.5 | 7.7±2.2 | 7.2±1.9 | 8.3±1.3 | 7.3±2.3 | N/A |
| Co-morbidities | 7 | 8 | 5 | 7 | 27 | N/A |
| Respiratory care (oxygen) | 0 | 0 | 6 | 8 | 14 | N/A |
| Mechanical ventilation | 0 | 0 | 0 | 1 | 1 | N/A |
| Death | 0 | 0 | 0 | 3 | 3 | N/A |

Note: Comorbidities: coronary heart disease, hypertension, cerebrovascular, stroke, neoplastic, chronic lung, liver, and renal diseases; diabetes mellitus; autoimmune disorders; and allergic diseases.

Complications: clinico-radiographic pneumonia, bronchitis, acute exacerbation of chronic pulmonary diseases, acute cardiovascular/cerebrovascular events, and renal and metabolic derangements.

NC: sex and aged paired healthy individuals without any known medical conditions.

**Supplemental Table 3. Demographics and characteristics of patients infected with Omicron SARS-CoV-2 and corresponding healthy controls.**

| Characteristics | Mild  (N=54) | Moderate  (N=9) | Severe  (N=10) | Critical  (N=35) | Total  (N=108) | NC  (N=53) |
| --- | --- | --- | --- | --- | --- | --- |
| Sex, male/female | 24/30 | 4/5 | 8/2 | 18/17 | 54/54 | 22/31 |
| Age (years, mean±SD) | 53.0±20.0 | 70.9±12.9 | 72.5±3.1 | 68.0±14.8 | 60.8±18.9 | 45.53±14.19 |
| Complications | 2 | 2 | 7 | 32 | 43 | N/A |
| Anti-virus therapy | 3 | 3 | 10 | 35 | 51 | N/A |
| Log_10_ viral load on admission (mean±SD in copies/mL) | 8.9±1.9 | 10.3±0.9 | 9.4±0.7 | 8.9±1.6 | 9.1±1.7 | N/A |
| Co-morbidities | 31 | 6 | 4 | 18 | 59 | N/A |
| Respiratory care (oxygen) | 0 | 0 | 7 | 35 | 42 | N/A |
| Mechanical ventilation | 0 | 0 | 0 | 4 | 4 | N/A |
| Death | 0 | 0 | 0 | 6 | 6 | N/A |

Note: Comorbidities: coronary heart disease, hypertension, cerebrovascular, stroke, neoplastic, chronic lung, liver, and renal diseases; diabetes mellitus; autoimmune disorders; and allergic diseases.

Complications: clinico-radiographic pneumonia, bronchitis, acute exacerbation of chronic pulmonary diseases, acute cardiovascular/cerebrovascular events, and renal and metabolic derangements.

NC: sex and aged paired healthy individuals without any known medical conditions.


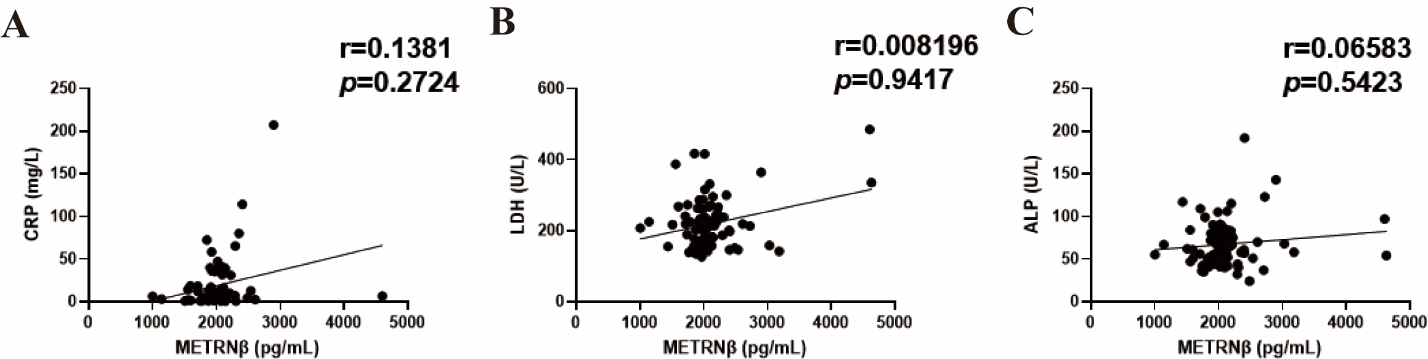


**Figure S1.** Correlation of circulating MERTNβ levels with biochemical parameters in the prediction cohort. Circulating MERTNβ concentrations of non-Omicron-SARS-CoV-2 infected COVID-19 patients in the prediction cohort measured by ELISA. Spearman’s correlation coefficient was used between MERTNβ and (A) CRP (n=65), (B) LDH (n=82), and (C) ALP (n=88) for correlation analysis. CRP: C-reactive protein, LDH: lactate dehydrogenase, ALP: alkaline phosphatase.
